# CD4^+^ T‐Cell‐Intrinsic IL‐6 Is Critical for Th17 Differentiation and Dampened Responsiveness to CD8^+^ T Cell‐Mediated Suppression

**DOI:** 10.1002/eji.70150

**Published:** 2026-02-23

**Authors:** Chakrapani Vemulawada, Kshitija Kale, Michael P. Crawford, Nitin J. Karandikar

**Affiliations:** ^1^ Department of Pathology University of Iowa Health Care and Iowa City VA Medical Center Iowa City Iowa USA

**Keywords:** CRISPR‐gene KO, effector suppression, IL‐6/IL6R, T‐cell intrinsic, Th17 cell

## Abstract

This study investigates the role of T‐cell‐intrinsic IL‐6 in Th17 differentiation and effector resistance. Using CRISPR‐Cas9‐mediated *Il6* knockdown in primary human CD4^+^ T‐cells, we demonstrate that T‐cell‐produced IL‐6 is crucial for the optimal development of Th17 cells. Lack of T‐cell‐endogenous *Il6* resulted in impaired IL‐17A production as well as significantly increased responsiveness to immune suppression. Importantly, these effects could not be reversed by the addition of exogenous IL‐6, likely due to interference with *Il6R* expression in the absence of endogenous *Il6*, with consequent reduction in STAT3 phosphorylation. Blockade of IL‐6 receptor replicated the functional effects of *Il6* knockdown and revealed a feedback loop between *Il6* and *Il6R* expression. Of note, IL‐21 was able to bypass the need for IL‐6 in both Th17 differentiation and the development of resistance. Our findings emphasize T‐cell‐endogenous IL‐6 as a critical cytokine in Th17 cell biology and highlight IL‐6 and IL‐21 as potential immunotherapeutic targets.

## Graphical abstract

1. Normal state: T‐cell‐endogenous IL‐6 stabilizes IL6R and promotes STAT3 activation, functional Th17 differentiation with resistance to immune suppression. 2. *Il6*‐KO: Loss of T‐cell endogenous IL‐6 destabilizes IL6R expression, leading to suboptimal Th17 and greater susceptibility to suppression, despite availability of exogenous IL‐6. 3. IL‐21 bypasses the requirement for endogenous IL‐6 by restoring STAT3 signaling and fully rescuing Th17 differentiation and effector resistance.

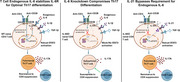

## 1 Introduction

The immune system operates as a highly intricate network of cells and molecules that collectively defend the body from pathogens while maintaining tolerance to self‐antigens [1, 2, 3]. Among the various immune cell subsets, Th17 cells, a subset of CD4^+^ T helper cells, are particularly important in mediating inflammation and immune responses to extracellular pathogens, as well as contributing to the pathology of several autoimmune diseases [4, 5]. Th17 cells are defined by their production of cytokines such as IL‐17A, IL‐17F, IL‐21, and IL‐22, which are involved in promoting inflammation and driving autoimmune processes [4, 5, 6].

The differentiation of Th17 cells from naive CD4^+^ T‐cells is tightly regulated by a complex network of cytokines, with IL‐6 playing a pivotal role in this process [6]. IL‐6 is a pleiotropic cytokine produced by various cell types, and mostly studied in macrophages, fibroblasts, endothelial cells, and other non‐T‐cells. It is crucial in regulating immune responses, inflammation, and hematopoiesis [7]. IL‐6 signals through its receptor, IL‐6R, and activates intracellular pathways, particularly the JAK/STAT pathway, leading to the expression of key transcription factors such as *Stat3* [8, 9]. While the soluble form of IL‐6R (sIL‐6R) can also activate these pathways in cells lacking the membrane‐bound IL‐6R, the direct signaling of IL‐6R on T‐cells plays a critical role in Th17 cell differentiation [10, 11]. Recent work shows that TCR‐Lck/Fyn signaling elicits IL‐6–independent STAT3 activation, which cooperates with cytokine inputs to shape Th17 programs [12].

The differentiation of Th17 cells is typically influenced by cytokines such as IL‐6, IL‐1β, TGF‐β1, IL‐21, and IL‐23, with each contributing to distinct aspects of Th17 development [13]. Notably, IL‐6 provides the primary signal for initiating Th17 differentiation, while IL‐1β enhances this process by activating pathways like NF‐κB and MAPK, promoting *RORγt* expression, a critical transcription factor for Th17 cells [14]. Understanding how different combinations of these cytokines influence the development of Th17 subsets is essential for elucidating the mechanisms underlying immune responses and inflammation in autoimmune diseases. We have previously shown that Th17 cells are resistant to immune suppression [15], a characteristic that may contribute to their persistence and pro‐inflammatory activity in autoimmune conditions. This effector resistance is thought to be linked to the cytokine profile and transcriptional regulation of Th17 cells.

We and others have also observed the production of IL‐6 by T‐cells themselves [15, 16, 17, 18]. However, T cells as a source of IL‐6 have been largely underappreciated. Whereas prior studies have established the importance of IL‐6R‐mediated T‐cell‐intrinsic signaling in the development of Th17 cells and overcoming immune suppression [16], the role of T‐cell‐endogenous IL‐6 remains poorly understood. In this study, we focused on understanding the specific contributions of T‐cell‐endogenous IL‐6 in the development and function of human Th17 cells, in the context of various cytokine milieus that promote Th17 differentiation. Using CRISPR/Cas9‐mediated knockdown of *Il6* in human naïve CD4 T‐cells (and antibody‐mediated blockade of IL‐6R), we demonstrate the critical role of T‐cell‐intrinsic IL‐6 in Th17 differentiation and effector resistance. We believe that our work builds on prior findings, while revealing some novel aspects of this fundamental biology, potentially opening new avenues for immunotherapeutic intervention strategies.

## 2 Results

### 2.1 Deletion of CD4 T‐Cell‐Endogenous *Il6* Significantly Diminishes IL17 Production and Reverses Effector Resistance

The differentiation of Th17 cells from naïve CD4+ T‐cells is strongly influenced by combinations of cytokines, most notably IL‐6, IL‐1β, TGF‐β1, IL‐21, and IL‐23 [19, 20]. Exogenous IL‐6 is known to act in concert with TGF‐β1 to drive the differentiation of naïve CD4+ T‐cells into Th17 cells [21, 22]. Importantly, T‐cells themselves are capable of expressing *Il6* [15, 17, 18]. However, the role or necessity of this T‐cell‐endogenous IL‐6 is poorly understood and was the focus of these studies.

We first confirmed prior observations that purified T cells produce IL‐6 by magnetically sorting naïve CD4 T‐cells and activating them in vitro using anti‐CD3/anti‐CD28. Cells and supernatants were harvested at various longitudinal time points, followed by measurement of *Il6* mRNA in the cells as well as IL‐6 protein in the supernatants. Our findings confirmed the ability of T‐cells to produce IL‐6 (Figure S2).

**FIGURE 1:**
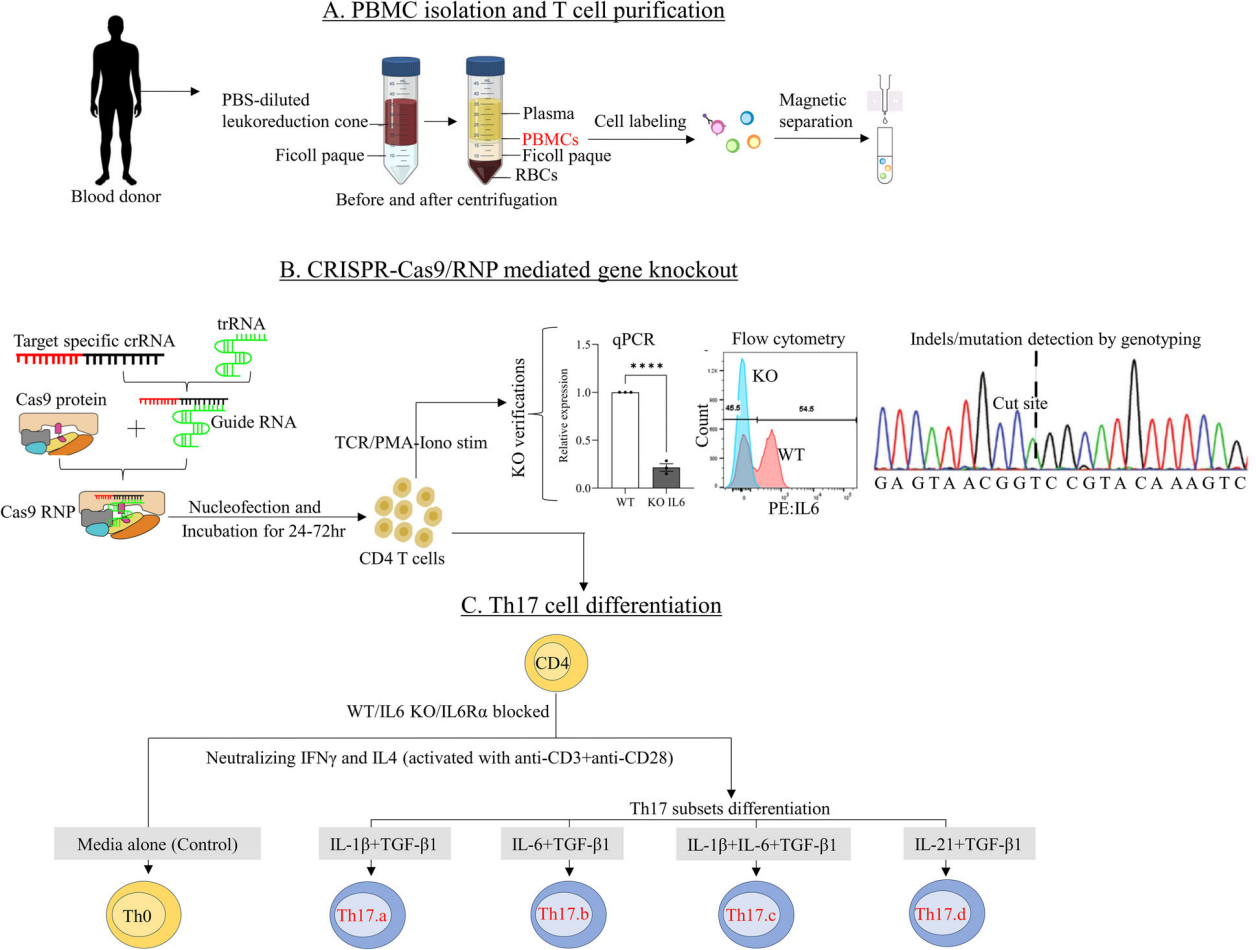
**Schematic diagram outlining the overall experimental approach**. (A) PBMCs were isolated from healthy donor leukoreduction cones, and naïve CD4^+^ T cells were purified by magnetic separation. (B) Naïve CD4^+^ T cells were used for CRISPR‐Cas9 knockout experiments with gene‐specific gRNAs, and KO efficiency was assessed after nucleofection. (C) Cells were then subjected to Th17 differentiation under four cytokine conditions (Th17.a, Th17.b, Th17.c, and Th17.d) in different combinations of TGF‐β1, IL‐6, IL‐1β, or IL‐21, with anti‐CD3/CD28 activation for 7 days.

To directly test the function of T‐cell‐intrinsic IL‐6, we knocked down *Il6* expression in magnetically enriched naive CD4 T‐cells, using the CRISPR‐Cas9 RNP method (Figure 1), as published previously [23, 24, 25]. Post‐knockdown viability and *Il6* mRNA expression were evaluated, demonstrating a significant reduction of *Il6* expression compared with SHAM‐KO controls (Figure S3A), while maintaining similar viability between *Il6*‐KO and SHAM‐KO controls (Figure S3B), matching our recent experience in using this method in primary human CD8 T‐cells [25].

**FIGURE 2:**
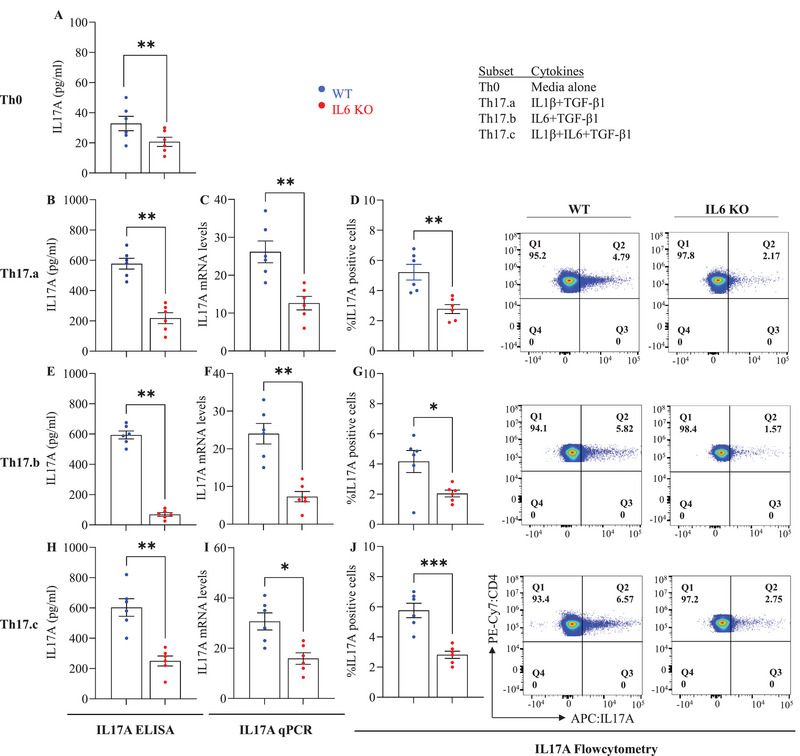
***Il6–*deficient Th17 cells show reduced IL‐17A production**. Control and *Il6* KO cells were cultured under the indicated Th17 differentiation conditions. On day 7, cytokine levels were assessed by ELISA (A, B, E, H), qRT‐PCR (C, F, I), and intracellular flow cytometry (D, G, J), with representative dot plots shown in the last two columns. Each dot represents an individual donor; error bars indicate SEM. Data are from three independent experiments (*n* = 6 per group). Statistical significance was determined using unpaired Student's *t*‐test (*p ≤ 0.05; **p ≤ 0.01; ***p ≤ 0.001; **p ≤ 0.0001).

In the first set of KO experiments, the *Il6* KO naive CD4 T‐cells (and the SHAM‐KO control cells) were cultured for 7 days in 4 distinct polarizing conditions that included media alone (Th0 controls) and the 3 indicated combinations of IL‐1β, IL‐6 and TGF‐β1 (Figure 2; Figure S4). Maintenance of *Il6* knockdown over the 7 days of culture was confirmed by qPCR assays on day 7 of these cultures (Figure S4A–C), as well as measurement of IL‐6 cytokine in supernatants by enzyme‐linked immunosorbent assay (ELISA), restricted to conditions Th0 and Th17.a, where no exogenous IL‐6 was added (Figure S4D,E). All conditions showed significant *Il6* knockdown, including the Th0 condition, which produced small amounts of IL‐6 cytokine to begin with, confirming robust knockdown using our approach.

Next, on day 7 of the differentiation cultures, we quantified *Il17A* expression using three distinct and complementary approaches: [1] qPCR for mRNA expression, [2] ELISA assays on culture supernatants, and [3] intracellular cytokine flow cytometry following a 4 h stimulation with PMA+ionomycin+brefeldin‐A. Th0 cells showed very low IL‐17A production (as expected), which was further diminished following the knockdown of T‐cell‐intrinsic IL‐6 (Figure 2A). In condition Th17.a, where no IL‐6 was added exogenously, the knockdown of T‐cell‐intrinsic IL‐6 resulted in a significant reduction in IL‐17A production, as measured by all three assays (Figure 2B,D), indicating the need for T‐cell‐intrinsic IL‐6 in this process. Of significant note, even the addition of exogenous IL‐6 [culture conditions Th17.b (IL‐6^+^TGF‐β1) and Th17.c (IL‐6^+^IL1‐β^+^TGF‐β1)] was not able to compensate for the deficit of T‐cell‐intrinsic IL‐6, as evidenced by the dramatic reduction of IL‐17A production across all assays (Figure 2E–J). Collectively, these results indicate that T‐cell‐endogenous IL‐6 plays a crucial role in the generation of Th17 T‐cells from naive CD4 T‐cells and that this role cannot be fully assumed by exogenous IL‐6.

### 2.2 Th17 Subsets Lacking Endogenous IL‐6 Exhibited Significantly Reduced Effector Resistance to Suppression

**FIGURE 3:**
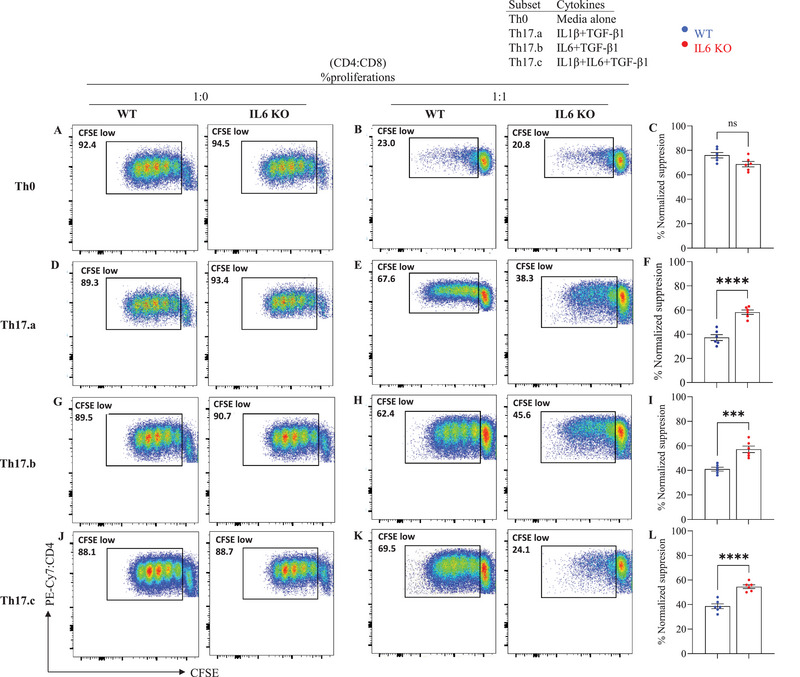
***Il6* KO Th17 subsets show altered responsiveness to CD8^+^ T cell–mediated suppression**. WT and *Il6* KO CD4^+^ T cells were differentiated under Th0 or the indicated Th17 conditions for 7 days, labeled with CFSE, and then cultured with or without CD8^+^ T cells for suppression assays. Proliferation was assessed by flow cytometry. Representative CFSE plots are shown for Th0, Th17.a, Th17.b, and Th17.c cultures (A, D, G, J: without CD8^+^ T cells; B, E, H, K: with CD8^+^ T cells). Bar graphs (C, F, I, L) summarize normalized percent suppression (mean ± SEM). Each point represents an individual donor; data are from three independent experiments (*n* = 6 per group). Statistical significance was determined using paired or unpaired Student's *t*‐test (*p ≤ 0.05; **p ≤ 0.01; ***p ≤ 0.001; **p ≤ 0.0001).

We have previously shown that naïve CD4 T‐cells that are in vitro‐differentiated into Th17 cells acquire resistance to suppression by CD8 T‐cells, partly mediated by the direct autocrine/paracrine action of IL‐17 on the T‐cells [15]. We have also validated these in vitro suppression assays for in vivo relevance using the xenogeneic‐GVHD models [26]. We therefore utilized these assay systems to test effector resistance in the Th17.a, Th17.b and Th17.c subsets (and control Th0 cells) that were generated from *Il6*‐deficient CD4 T‐cells (versus *Il6*‐replete controls). Thus, on day 7 of differentiation cultures, cells were stained with CFSE and used as responder cells in flow cytometric suppression assays, with autologous bulk CD8 T‐cells as the suppressors, as described previously [15, 25, 27]. The suppression assay results are presented in Figure 3. First, focusing on the control cell populations (“WT”), we observed the expected effector resistance in the three Th17 conditions compared with the Th0 condition (left dot plots in Figure 3B,E,H,K and left bars in Figure 3C,F,I,L).

Knockdown of *Il6* in the Th0 condition did not significantly affect their suppressibility to suppression (Figure 3A–C). However, *Il6* KO Th17.a, Th17.b and Th17.c subsets showed significantly greater susceptibility to suppression by autologous CD8 T‐cells compared with the control Th17 cells (Figure 3D–L). These data demonstrate the key role of T‐cell‐intrinsic IL‐6 in the acquisition of effector resistance by these Th17 subsets. Again, exogenous IL‐6 was unable to compensate for the lack of T‐cell‐endogenous IL‐6 (as indicated by the Th17.b and Th17.c subsets).

### 2.3 Blockade of IL‐6R on CD4^+^ T‐Cells Results in Reduced Th17 Differentiation and Mitigated Effector Resistance

It is fairly well established that exogenous IL‐6 signals through the IL‐6 receptor (IL‐6R), which can be membrane‐bound or soluble [28], and that these IL‐6R‐mediated downstream signaling pathways [29, 30] are essential for Th17 differentiation, through the expression of transcription factors like *RORγt*, which are central to the Th17 phenotype [30, 31]. However, the dynamics of IL‐6R signaling in the absence of exogenous IL‐6 have not been studied. We hypothesized that T‐cell‐intrinsic IL‐6 would require IL‐6R‐mediated signaling to effect Th17 differentiation and effector resistance. To test this hypothesis, we focused on culture conditions that did not contain exogenously provided IL‐6 (i.e, Th0 and Th17.a) and employed an anti‐IL6Rα antibody to block the IL‐6R. These IL6Rα‐blocked CD4 T‐cells were then cultured in Th0 or Th17.a condition.

**FIGURE 4:**
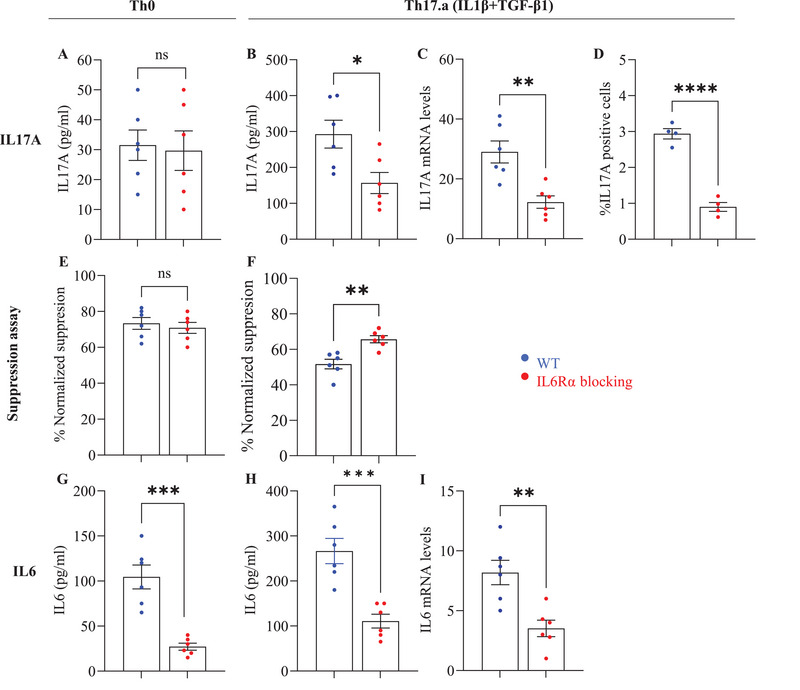
**Blockade of IL‐6Rα impairs Th17 development and alters responsiveness to CD8^+^ T cell–mediated suppression**. Naïve CD4^+^ T cells were cultured under Th0 or Th17.a conditions in media alone (WT) or with anti–IL6Rα antibody. On day 7, IL‐17A expression was measured by qPCR, ELISA, and flow cytometry (A–D). Cells were also subjected to CD8^+^ suppression assays (E, F), and IL–6 expression was assessed at the protein and mRNA level (G–I). Data are from three independent experiments (*n* = 6 per group); each point represents an individual donor. Error bars indicate SEM. Statistical analysis was performed using paired or unpaired Student's *t*‐test (*p ≤ 0.05; **p ≤ 0.01; ***p ≤ 0.001; **p ≤ 0.0001).

On day 7 of the differentiation cultures, *Il17A* was quantified using qPCR for mRNA, ELISA for supernatant cytokine, and flow cytometry for intracellular cytokine. In the Th0 control condition, there was no significant change in the minimal IL‐17A production observed from both WT and IL6Rα‐blocked cultures (Figure 4A). However, the *Il17A* mRNA expression, IL‐17A cytokine production, and the frequency of IL‐17A‐positive cells were significantly decreased in the IL6Rα‐blocked Th17.a subset, relative to the WT Th17.a subset (Figure 4B–D). Further, to assess the role of IL‐6/IL6Rα in Th17 resistance to immunosuppression, ex vivo suppression assays were performed using IL6R‐blocked and control Th17.a subsets along with the Th0 condition. Whereas suppression of Th0 cells was unaffected by IL‐6R blockade (Figure 4E), IL6R‐blocked Th17.a cells showed significantly increased suppression by autologous CD8 T‐cells compared with WT Th17.a cells (Figure 4F). Collectively, these results indicate that IL‐6R signaling by T‐cell‐endogenous IL‐6 is critical for the optimal development of Th17 cells and effector resistance.

Since blockade of IL‐6R resulted in reduction of Th17 differentiation and effector resistance, we wondered whether there was any feedback loop between T‐cell‐endogenous IL‐6 production and IL6R‐mediated signaling. Thus, we measured the IL‐6 cytokine and mRNA levels in Th0 and Th17.a condition with and without IL‐6R blockade. We observed that blockade of IL‐6Rα resulted in the reduction of IL‐6 protein as well as *Il6* message by these T‐cells (both Th0 and Th17.a) (Figure 4G–I). These findings suggest an interesting autocrine/paracrine feedback loop between T‐cell‐produced IL‐6 and IL‐6R signaling that plays a critical role in Th17 differentiation.

### 2.4 IL‐21 Bypasses the Requirement of T‐Cell‐Intrinsic IL‐6/IL‐6R Signals in Th17 Differentiation and Acquisition of Effector Resistance

Th17 cells can be generated in various cytokine milieus. One such cytokine that can push cells toward the Th17 lineage is IL‐21 [32, 33]. In non‐T immune cells, IL‐21 signaling has been shown to bypass the IL‐6 signals by directly activating downstream pathways [34, 35, 36]. Unpublished data from our group shows that Th17 cells differentiated in the presence of IL‐21+TGF‐β1 exhibit high resistance to immunosuppression. We therefore decided to test whether the use of this combination (condition “Th17.d”) would also bypass the requirement of T‐cell‐intrinsic IL‐6/IL‐6R signaling. Thus, *Il6* KO or IL6Rα‐blocked naive CD4 T‐cells were separately differentiated in Th17.d conditions for 7 days, followed by assessment for IL‐17A production as well as cell resistance in suppression assays.

**FIGURE 5:**
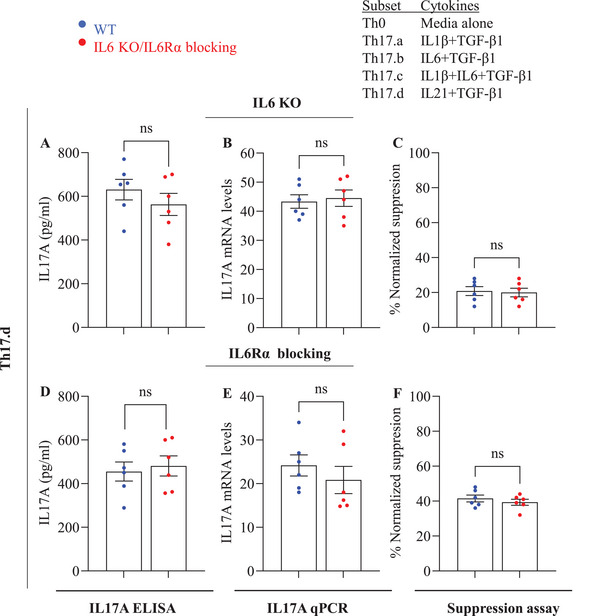
**Th17 cultures containing IL‐21+ TGF‐β1 (Th17.d) develop independently of T cell–intrinsic IL‐6**. WT or *Il6* KO CD4^+^ T cells were differentiated under Th17.d conditions for 7 days, and *Il17A* expression was assessed by ELISA (A) and qPCR (B). Responsiveness to CD8^+^ T‐cell‐mediated suppression was evaluated in coculture assays (C). In parallel, WT cells were cultured with or without anti–IL6R antibody and subjected to the same functional readouts (D–F). Data are from three independent experiments (*n* = 6 per group); each point represents an individual donor. Error bars indicate SEM. Statistical analysis was performed using paired or unpaired Student's *t*‐test (*p ≤ 0.05; **p ≤ 0.01; ***p ≤ 0.001; **p ≤ 0.0001).

Once again, significant long‐term *Il6* knockdown was confirmed in the Th17.d conditions using qPCR as well as ELISA assays (Figure S5A,B). Importantly, when *Il6*‐KO or IL6R‐blocked cells were grown in Th17.d conditions, we did not see any reduction in *Il17A* mRNA expression or protein production (Figure 5A,B,D,E). In addition, neither of these interventions affected the ability of Th17.d cells to attain significant resistance to immune suppression (Figure 5C,F). Collectively, these data show that IL‐21 obviates the need for IL‐6 signaling in Th17 differentiation and effector resistance.

We also measured *Il6* message and protein in these conditions and found that IL‐6Rα blockade resulted in a reduction of IL–6 production, similar to the feedback loop from other culture conditions (Figure S5C,D). Despite this reduction, Th17.d cells showed robust Th17 differentiation and effector resistance to suppression.

### 2.5 *Il6* Knockout or IL‐6Rα Blockade Alters Cytokines and Transcription Factors Expression in Th17 Differentiation

The differentiation of Th17 T‐cells is regulated by a specific set of signature cytokines and transcription factors, which are essential for the development and stability of these cells [37, 38]. To explore whether disruption of T‐cell‐intrinsic IL‐6 signaling (via *Il6* KO or IL‐6Rα blockade) alters the expression of key Th17 signature molecules, we tested the mRNA expression of several implicated genes in the four distinct Th17 subsets: Th17.a, Th17.b, Th17.c, and Th17.d. These subsets were generated from *Il6* KO and IL6R‐blocked CD4 T‐cells. The key molecules tested included *RORγt*, *T‐bet*, *GM‐CSF*, and *Il1β*, all of which are critical for Th17 differentiation and function.

**FIGURE 6:**
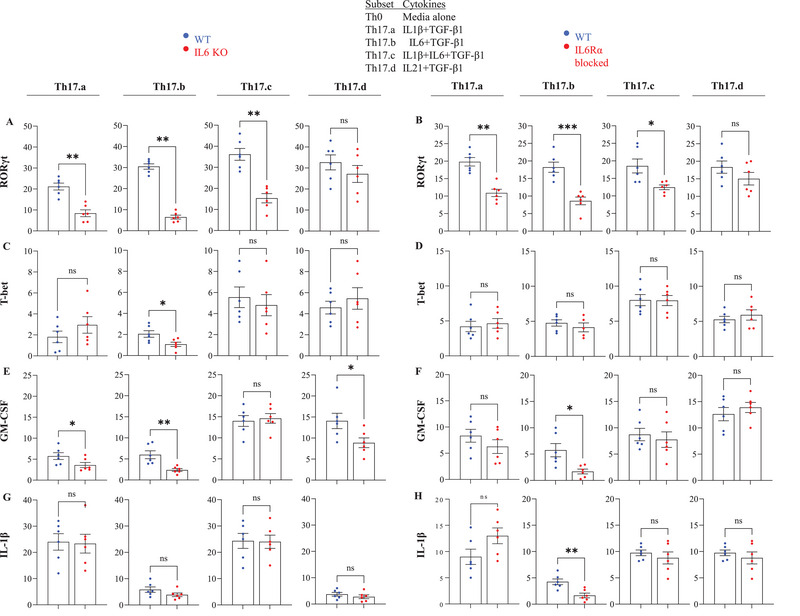
**Cytokines and transcription factors are differentially regulated in *Il6* KO and IL6Rα–blocked Th17 subsets**. On day 7 of Th17 differentiation, relative mRNA expression of cytokines and transcription factors (*RORγt*, *T‐bet*, *GM‐CSF*, *Il1β*) was quantified in *Il6* KO and IL6Rα‐blocked Th17.a, Th17.b, Th17.c, and Th17.d subsets compared with WT controls (A–H). Data are from three independent experiments (*n* = 6 per group); each point represents an individual donor. Error bars indicate SEM. Statistical analysis was performed using paired or unpaired Student's *t*‐test (*p ≤ 0.05; **p ≤ 0.01; ***p ≤ 0.001; **p ≤ 0.0001).

Our results show that *Il6* KO and IL‐6Rα blockade have subset‐specific effects on the expression of these factors. In most Th17 subsets (Th17.a, Th17.b, Th17.c), IL‐6 signaling was critical for *RORγt* expression, with the notable exception of Th17.d, where no significant changes were observed, likely due to *Il6*‐independent action (Figure 6A,B). *T‐bet* expression was notably reduced in the *Il6* KO Th17.b subset but remained unaffected in other subsets (Figure 6C,D). *GM‐CSF* expression was decreased in *Il6* KO Th17.a, Th17.b, and Th17.d subsets, but not in Th17.c, indicating IL‐6's importance for GM‐CSF production in specific subsets (Figure 6E). IL‐6Rα blockade did not alter *GM‐CSF* expression in Th17.a, Th17.c, and Th17.d subsets, but reduced it in Th17.b, similar to the *Il6* KO Th17.b subset (Figure 6F). *Il1β* expression was unaffected by *Il6* KO in most subsets, except for a reduction in Th17.b subset with IL‐6Rα blockade (Figure 6G,H). These findings highlight the complex and subset‐specific role of IL‐6 signaling in regulating Th17 differentiation and the expression of key cytokines and transcription factors.

We also investigated the impact of *Il6* KO on the expression of other T‐cell cytokines and transcription factors in the four Th17 subsets (Figure S6). In the *Il6 KO* Th17.b subset, *IFN‐γ* expression was significantly reduced, while no changes were observed in other Th17 subsets (Figure S6A). *Tgfβ1* expression remained similar between *Il6 KO* and WT Th17 subsets (Figure S6B). IL‐6 signaling was crucial for *Jak1* and *Jak2* expression in Th17.a, Th17.b, and Th17.c subsets, but not in Th17.d, where IL‐21 may compensate for the loss of IL‐6 signaling (Figure S6C,D). *Il6* KO Th17 subsets developed without IL‐21 showed reduced *Il17F*, *Il22*, and *CCR6* expression compared with WT, whereas Th17.d cells cultured with IL‐21 displayed no significant differences from their WT counterparts (Figure S6H–J). *FoxP3* and *Il10* were upregulated in the *Il6* KO Th17.b subset, suggesting a shift toward a more regulatory‐associated phenotype, while *Il5* expression remained low across all subsets, indicating that IL‐6 does not drive Th2 differentiation (Figure S6E–G). These findings highlight IL‐6's critical role in regulating Th17 differentiation and their functional diversity, especially in promoting regulatory‐associated characteristics in specific subsets.

### 2.6 T‐Cell‐Intrinsic IL‐6 Modulates Th17 Differentiation Through IL‐6Rα Stabilization and Generation of pSTAT3

**FIGURE 7:**
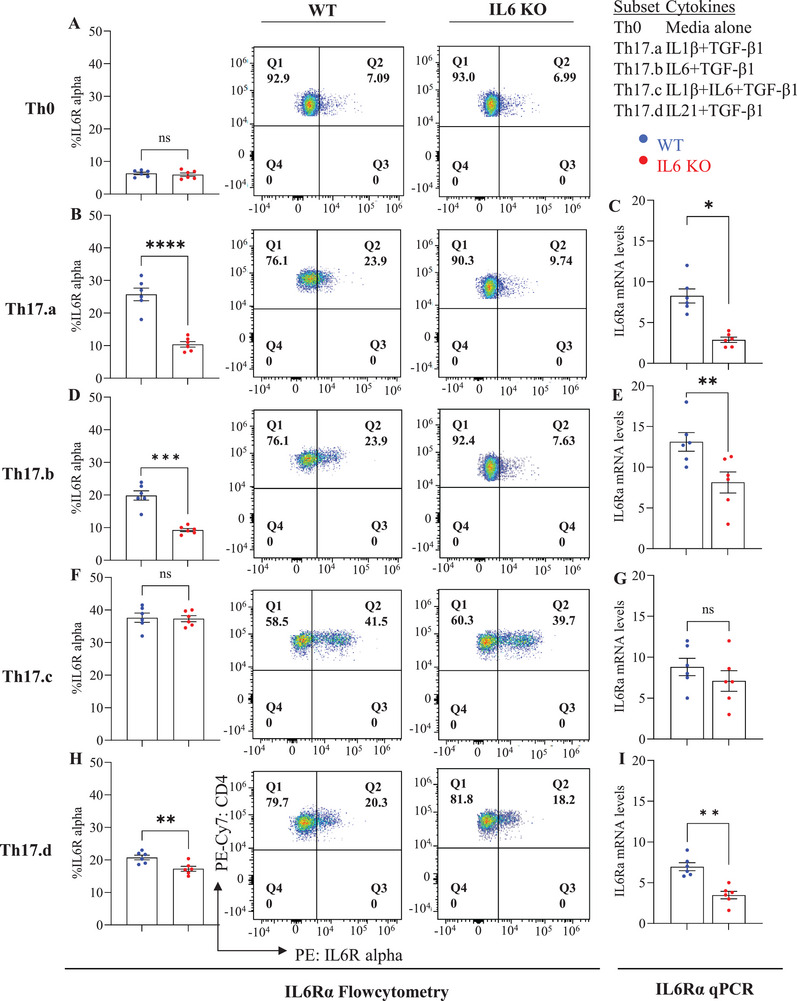
**T cell–intrinsic IL6 supports optimal *Il6Rα* expression**. Naïve CD4^+^ T cells from WT and *Il6* KO conditions were differentiated under Th17.a, Th17.b, Th17.c, and Th17.d cultures, and IL‐6Rα expression was measured by flow cytometry and qRT‐PCR. Flow cytometry plots and bar graphs show IL‐6Rα^+^ CD4^+^ T cells in Th0 and Th17 subsets (A, B, D, F, H), and qPCR results show *Il6Rα* mRNA levels (C, E, G, I). Data are from three independent experiments (*n* = 6 per group); each point represents an individual donor. Error bars indicate SEM. Statistical analysis was performed using paired or unpaired Student's *t*‐test (*p ≤ 0.05; **p ≤ 0.01; ***p ≤ 0.001; **p ≤ 0.0001).

We further dissected the mechanism of T‐cell‐intrinsic IL‐6 involvement in Th17 differentiation. Based on the evidence of an IL‐6/IL‐6R feedback loop (Figure 4; Figure S5), we focused on the effect of T‐cell IL‐6 deficiency on IL‐6Ra and the associated STAT3 phosphorylation. Thus, we first analyzed *Il6R* expression in Th17 subsets (Th17.a, Th17.b, Th17.c, and Th17.d) from *Il6* KO and WT CD4 T‐cells, by quantifying both the α and β subunits. On day 7, qPCR and flow cytometry revealed no significant differences in the expression of the β subunit (*Il6Rβ* or *gp130*) across these subsets, including Th0 controls (Figure S7). In contrast, *Il6Rα* expression was significantly decreased in Th17.a and Th17.b subsets, marginally reduced in the Th17.d subset, and not significantly altered in the Th17.c subset (Figure 7A–I).

**FIGURE 8:**
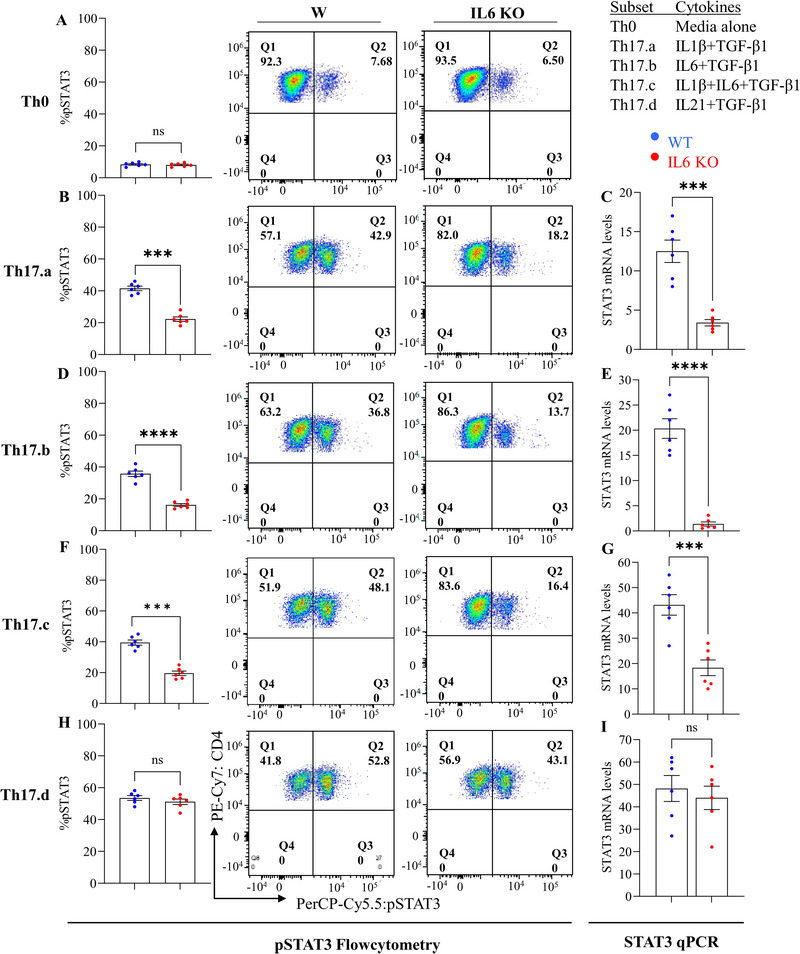
**Endogenous *Il6* deletion reduces STAT3 phosphorylation during Th17 differentiation**. Naïve CD4^+^ T cells from WT and *Il6* KO conditions were differentiated into Th17.a, Th17.b, Th17.c, and Th17.d subsets. On day 7, *Stat3* mRNA expression and the frequency of phosphorylated STAT3^+^ (pSTAT3) CD4^+^ T cells were measured by qPCR and flow cytometry. Representative plots and bar graphs show pSTAT3^+^ CD4^+^ T cells in Th0 and Th17 subsets (A, B, D, F, H), while qPCR results display *Stat3* mRNA expression (C, E, G, I). Data are from three independent experiments (*n* = 6 per group); each point represents an individual donor. Error bars indicate SEM. Statistical analysis was performed using paired or unpaired Student's *t*‐test (*p ≤ 0.05; **p ≤ 0.01; ***p ≤ 0.001; **p ≤ 0.0001).

Next, we compared STAT3 phosphorylation levels and mRNA expression in *Il6* KO and WT CD4 T‐cells from these conditions. Flow cytometry and qPCR revealed that in Th0 media controls, STAT3 phosphorylation was similar in both *Il6* KO and WT‐cells (Figure 8A), suggesting IL‐6 is not a primary driver of STAT3 activation in naïve‐derived Th0 cells. However, in Th17.a, Th17.b, and Th17.c subsets, STAT3 phosphorylation, and mRNA expression were significantly reduced in *Il6* KO cells compared with WT (Figure 8B–G). In contrast, knocking out *Il6* did not affect STAT3 phosphorylation in the IL‐21‐mediated Th17.d cells (Figure 8H,I). Similarly, *Irf4* expression was significantly reduced in *Il6* KO Th17.a, Th17.b, and Th17.c subsets compared with WT, while no difference was observed in the IL‐21–supplemented Th17.d subset (Figure S6K).

A similar pattern of *Stat3* mRNA expression was observed in the context of IL‐6Rα blockade, with significant changes in the Th17.a, Th17.b, and Th17.c subsets, but not in Th17.d cells (Figure S8). Interestingly, the change in Th17.c cells was less robust compared with that in Th17.a and Th17.b. These results indicate that T‐cell‐intrinsic IL‐6 signaling is essential for STAT3 activation in Th17.a, Th17.b, and Th17.c subsets, while Th17.d differentiation, driven by IL‐21+TGF‐β1, is independent of IL‐6 signaling, suggesting a cytokine‐specific regulation of STAT3 across Th17 subsets.

Collectively, there were some notable differences between the different Th17 subsets. The Th17.a condition did not have exogenous IL‐6 and relied entirely on an intrinsic source of IL‐6. The Th17.b condition had exogenous IL‐6 provided, but the robust downregulation of IL‐6R in the *Il6*‐KO cells might explain the lack of rescuing IL‐6 signaling. The IL‐21‐containing Th17.d condition completely bypassed the need for IL‐6. However, the Th17.c condition showed an interesting dynamic, by maintaining its *Il6R* expression (Figure 7), perhaps driven by exogenously provided IL‐1β. Despite this, these cells did not show optimal STAT3 phosphorylation, IL17 production, or effector resistance, again underscoring the key role for T‐cell‐endogenous IL‐6 in this context.

## 3 Discussion

It is well established that Th17 cells are important in the immune response to infections and as mediators of disease [4, 19, 20, 39, 40]. The important role of IL‐6 in influencing the differentiation of naïve CD4+ T‐cells into Th17 cells is also widely acknowledged [6, 8, 10, 31, 41]. However, in these settings, IL‐6 is modeled as a cytokine produced by non‐T‐cells [18, 42, 43, 44] and is thus provided exogenously in Th17 differentiation cultures, with the idea that the exogenous cytokine signals through the IL‐6R and effectuates downstream pathways that result in Th17 differentiation.

We have recently shown that human Th17 cells, derived in vitro from purified naïve CD4^+^ T‐cells, are resistant to immune suppression, compared with their control Th0 counterparts [15], partly owing to IL‐17‐mediated autocrine/paracrine signaling. During our mechanistic evaluation of this finding, we also found a message for IL‐6 within the T‐cells [15, 45, 46]. Other studies have also demonstrated that T‐cells are capable of endogenously producing IL‐6 cytokine [10, 16, 47, 48, 49, 50]. However, T‐cell‐endogenous IL‐6 has been generally underappreciated/understudied, and its role is poorly understood. In the current study, we focused on the potential role of T‐cell‐produced IL‐6 and demonstrated that T‐cell‐endogenous IL‐6 is a critical driver of Th17 differentiation and effector resistance to suppression.

After confirming that activated CD4^+^ T‐cells are capable of producing IL‐6, we used CRISPR‐Cas9‐mediated *Il6* knockdown in primary CD4^+^ T‐cells, an approach we had previously used successfully to knockout other effector molecules in primary CD8^+^ T‐cells [25]. We confirmed the efficacy of the knockdown, with the *Il6* KO cells showing significant reduction in *Il6* mRNA and protein levels (Figures S3 and S4). Importantly, the absence of T‐cell‐endogenous IL‐6 led to diminished *Il17A* expression across various Th17 subsets, even in conditions where exogenous IL‐6 was provided. Thus, Th17.a, Th17.b, and Th17.c subsets showed significantly lower *Il17A* message in the absence of T‐cell‐intrinsic IL‐6 (Figure 2), further confirmed by ELISA and flow cytometry (Figure 2A,B,E,H,D,G,J) These results highlight the critical nature of intrinsic IL‐6 in supporting Th17 differentiation, a function that cannot be subsumed by externally produced IL‐6.

We further tested whether T‐cell‐intrinsic IL‐6 was also required by these Th17 cells in acquiring resistance to CD8^+^ T‐cell‐mediated suppression. Correlating with their suboptimal Th17 differentiation, all three subtypes (Th17.a, Th17.b, and Th17.c) also showed significantly increased susceptibility to CD8^+^ T‐cell‐mediated suppression, compared with their control, IL‐6‐replete counterparts. Once again, this highlighted that T‐cell‐intrinsic IL‐6 is crucial for the acquisition of effector resistance in these subsets, a role that cannot be performed by exogenous cytokine.

Prior studies have shown that signaling through the IL‐6R on CD4+ T‐cells is critical in Th17 differentiation [10, 16, 18, 49, 51]. Based on our results from the *Il6* KO experiments, we hypothesized that the T‐cell‐intrinsic IL‐6 would require signaling through the IL‐6R (likely in an autocrine manner) to effectuate Th17 differentiation and effector resistance. Thus, we conducted experiments using WT control cells (IL‐6‐replete) but cultured them in the presence of anti‐IL6Rα antibody. For these experiments, we used the Th0 and Th17.a culture condition, as they did not contain any exogenously added IL‐6. Blockade of IL‐6Rα resulted in significantly reduced IL‐17A production by Th17.a cells and an increased susceptibility to CD8 T‐cell‐mediated suppression (Figure 4B–D). These results demonstrate that T‐cell‐intrinsic IL‐6 signals through the IL‐6R to effectuate optimal Th17 differentiation and highlight the importance of an autocrine IL‐6/IL‐6R axis in this process.

IL‐21 has been shown to bypass the need for IL‐6 signaling in B cells and Th17 cell differentiation [10, 22, 32, 36, 40, 44, 52, 53, 54, 55]. We hypothesized that a similar dynamic would play out in the context of T‐cell‐intrinsic IL‐6 deficiency. Indeed, when IL‐21 was added to the culture condition (Th17.d), neither the *Il6* KO cells nor the IL6Rα‐blocked cells showed any reduction in IL‐17A production or altered responsiveness to immunosuppression (Figure 5A–F). These findings suggest that, similar to the findings in other types of CD4 T helper cells (Th1 and Th2), IL‐21 signaling also bypasses the need for Th17 cell‐intrinsic IL‐6/IL‐6R signaling, likely by directly activating downstream pathways. Furthermore, IL‐21's ability to induce IL‐17A production and resistance to immune suppression in the absence of IL‐6 or IL‐1β signaling underscores its potential as a key regulator of Th17 immunity in inflammatory and autoimmune conditions. Interestingly, IL‐21 is also produced by Th17 cells [34, 55, 56, 57, 58], and it would be important to delineate the role of Th17‐produced IL‐21 in future studies.

Our results demonstrate that IL‐6 is essential for the expression of key Th17 transcription factors and cytokines, particularly *RORγt*, *T‐bet*, and *GM‐CSF*, across most Th17 subsets. However, Th17.d cells showed minimal changes in these markers, likely due to IL‐6‐independent signaling through IL‐21 (Figure 6A,C,E,G). Notably, IL‐6Rα blockade and *Il6* KO had similar effects on *GM‐CSF* expression, with differential impacts across subsets, particularly in Th17.b (Figure 6B,D,F,H). In line with prior reports of T‐cell‐intrinsic IL‐1β, our Th17 subsets showed *Il1β* mRNA differences without protein quantification [59]. Additionally, IL‐6 signaling was found to regulate *Jak1* and *Jak2* expression in Th17.a, Th17.b, and Th17.c subsets, but not in Th17.d, further supporting the importance of IL‐6 in JAK/STAT signaling for Th17 differentiation (Figure S6C,D). We also observed a shift toward a more regulatory‐associated phenotype in *Il6* KO Th17.b cells, characterized by upregulation of *FoxP3* and *Il10* (Figure S6E,F). Our findings also suggest that IL‐21 can function as an alternative pathway to sustain Th17.d differentiation in the absence of T cell–intrinsic IL‐6. By maintaining expression of *Il17F*, *Il22*, and key surface receptors *CCR6* (Figure S6G–I). IL‐21 helps preserve a Th17‐like program, highlighting cytokine‐specific redundancy in Th17 regulation. This complex, subtype‐specific regulation of effector pathways will be a focus of future studies. Finally, our studies highlight an interesting, interactive dynamic between IL‐6 and its receptor. We found that blockade of IL‐6Rα resulted in a reduction of T‐cell‐intrinsic IL‐6 production (Figure 4). Moreover, knockdown of *Il6* also resulted in the reduced expression of *Il6Rα* in Th17.a and Th17.b conditions but did not affect expression of *gp130* (IL‐6Rβ) (Figure 6). This may explain, in part, why exogenously provided IL‐6 (in condition Th17.b) could not overcome the deficit of T‐cell‐intrinsic IL‐6, as it could not optimally signal the T‐cell. It is tempting to speculate that the function of the intrinsically expressed cytokine might be to stabilize the expression of *Il6Rα*, which can then combine with IL‐6Rβ and allow the cell to receive further signals from its environment. This is corroborated by our observation that *Stat3* expression and phosphorylation were also affected in these cells (Figure S8; Figure 8). Consistent with TCR‐driven STAT3 activation [12], TCR‐only (Th0) stimulation elicited comparable early pSTAT3 in WT and *Il6* KO CD4^+^ T cells, whereas IL‐6–dependent conditions (Th17.a/b/c) showed a selective reduction of pSTAT3 and *stat3* mRNA in *Il6* KO cells, confirming that our phenotype maps to loss of autocrine IL‐6 rather than off‐target disruption of TCR–Lck/Fyn signaling. This might reveal an interesting biology that may be generalizable across different cell types and cytokines and will be an important focus of our future studies.

However, there are also a couple of caveats to this in our findings. First, in condition Th17.c (that contained both IL‐1β and IL‐6), the *Il6* KO cells showed no differences in *Il6Rα* message (Figure 7F). Moreover, there was evidence of similar downstream activation of STAT3 in this condition. Yet, we did not observe optimal Th17 differentiation or acquisition of immune resistance in the absence of intrinsic IL‐6. Furthermore, in condition Th17.d (that contained IL‐21), the *Il6* KO cells did show reduced *Il6Rα* message and reduced STAT3 activation, but IL‐21 was able to bypass the need for these pathways and induced optimal Th17 differentiation and acquisition of resistance. These findings suggest that IL6–STAT3 signaling contributes to *Irf4* regulation in Th17 subsets, whereas IL‐21 supplementation can maintain *Irf4* expression independent of IL‐6. The complex interplay between IL‐1β‐, IL‐6‐, and IL‐21‐induced pathways will be important to understand for guiding interventional strategies. These findings align with in vivo observations from GVHD models showing that CD4^+^ T cell resistance to CD8^+^‐mediated suppression contributes to pathogenic responses, underscoring the biological relevance of our in vitro system [26].

In summary, this study demonstrates that IL‐6 produced endogenously by CD4^+^ T cells is an important driver of Th17 differentiation, critical for the development, maturation, and effector function of Th17 cells, including their altered responsiveness to immune suppression. At the same time, we also show that IL‐21 can bypass the intrinsic IL‐6 requirement by inducing Th17 differentiation and enhancing resistance to suppression through alternative pathways. These findings provide new insights into cytokine‐specific regulation of Th17 differentiation and identify potential targets for therapeutic intervention in immune‐mediated diseases.

## 4 Data Limitations and Perspectives

Detailed donor demographic data (age, sex) were not available, preventing assessment of potential demographic influences on IL‐6 signaling and Th17 differentiation. The focus of this study was the role of T‐cell endogenous IL‐6 in Th17 differentiation. One limitation is that we did not specifically study its role (or lack thereof) in Th1/Th2 differentiation, which would serve as an additional negative control. However, we assessed baseline phenotype, viability, and TCR‐driven activation under cytokine‐neutral (Th0) conditions and nontargeting CRISPR RNP controls, which serve as controls for lineage‐independent effects. We did not observe any effects of *Il6*‐knockdown on the ability of Th0 cells to produce IFNγ, T‐bet, or IL5, indicating that these other machineries were intact. Finally, our cell sorting was performed using magnetic bead separation and not flow cytometry (to favor greater viability following knockdown procedures). While we obtained highly enriched populations, we concede that there was low‐frequency (∼1%) contamination with CD3‐negative cells, which should be taken into account while interpreting the data. Importantly, by the time we obtained post‐nucleofection samples (either negative controls or actual *Il6* knockdown cells), we obtained 100% pure CD3^+^CD4^+^ T cells. In addition, one of our major observations was that extrinsically added IL‐6 did not compensate for the lack of T‐cell‐endogenous IL‐6, thus negating any effects from potential IL‐6 produced by contaminating cells.

## 5 Methods and Materials

### 5.1 Primary Cell Preparation and Bead Sorting of T Lymphocytes

PBMC from healthy subjects were isolated from deidentified leukocyte reduction system cones containing leukocyte‐rich blood from platelet donors at the University of Iowa, DeGowin Blood Center, as depicted in Figure 1. All donors were screened by the Blood Center according to standard eligibility criteria. Beyond that screening data, detailed demographic information was not available for inclusion in our dataset.

PBMC isolation was performed with Ficoll‐Paque (GE Healthcare) density gradient centrifugation and frozen in DMSO‐containing media for further use when not used immediately, as described in earlier studies [25]. Naive CD4^+^ T lymphocytes were then enriched from fresh or frozen PBMC by negative selection using the naive CD4^+^ T‐cell isolation kit II (Miltenyi Biotec, 130‐094‐131) following the manufacturer's instructions. Sort purities were routinely >94% by flow cytometric analysis (Figure S1). On the day of suppression assays, autologous bulk CD8^+^ cells were isolated from thawed PBMC (RPMI 1640 [Corning, 10‐040‐CV] with DNase at 10 KU/mL [Sigma‐Aldrich, D4513‐1vl]) using human CD8 MicroBeads by positive selection (Miltenyi Biotec,130‐045‐201), as shown previously [15, 25].

### 5.2 CRISPR‐Cas9‐Mediated *Il6* Knockdown

CRISPR‐Cas9‐based gene knockouts were performed as previously described in primary human CD8^+^ T‐cells [25], and similar approaches were applied to naïve CD4^+^ T‐cells in this study. Guide RNAs (gRNAs) targeting genes of interest were designed using online tools optimized for high on‐target activity and low off‐target potential, including Synthego (https://design.synthego.com/#/) and IDT (https://www.idtdna.com/site/order/designtool/index/CRISPR_PREDESIGN) [25, 60, 61, 62]. To enhance deletion efficiency, two or more gRNAs per gene locus were used where possible (Table S1). Designed crRNAs included 20‐nt target‐specific sequences followed by a PAM (NGG) motif and were manually selected based on high on‐target scores (>70), minimal predicted off‐targets, and preferential targeting of early exons. Chemically modified synthetic gRNAs were purchased from Synthego, including gene‐specific sequences and nontargeting controls. Ribonucleoprotein (RNP) complexes were formed by incubating TrueCut Cas9 Protein v2 (Thermo Fisher Scientific, A36499) with each gRNA at a 1:3 molar ratio. 10–15 × 10^6^ naïve CD4^+^ T‐cells were resuspended in P3 nucleofection solution (Lonza, V4XP‐3024), combined with the RNP complexes, and transferred into 100 µL cuvettes (4D‐Nucleofector X Kit L; Lonza). Electroporation was performed using a 4D‐Nucleofector Core Unit and X Unit (Lonza) with the EH100 pulse code. Mock‐transfected and nontargeting gRNA‐transfected cells were included as negative controls. Following nucleofection, cells were cultured in 2 mL of prewarmed XVIVO‐15 medium (Lonza) for at least 24 h before gene disruption analysis and subsequent Th17 differentiation assays (see Figure 1 for workflow).

### 5.3 Intracellular Cytokine and Cell Viability Staining

1 × 10^6^ cells of each gene KO and wild‐type/negative control CD4 T‐cells were stimulated for 4 h with the leukocyte activation mixture, with BD GolgiPlug (BD Biosciences, 550583). After activation, cells were washed twice in 1× PBS, followed by one wash in FACS buffer, and the cells were surface antibody‐stained as described previously [15, 25, 26]. For intracellular staining, cells were treated with a fixation/ permeabilization buffer (Invitrogen, 00‐5123‐43) and then incubated with target‐specific conjugated antibodies diluted in permeabilization buffer (Invitrogen, 00‐8333‐56) and analyzed by flow cytometry (Cytek Aurora). Data were analyzed with FlowJo software (version 10.7.1). To determine the extent of cell survival and cell death, CD4 T‐cells were stained with a live/dead dye (Ghost Dye Violet 450 viability dye, Tonbo Biosciences, 13‐0863‐T100).

### 5.4 Total RNA Isolation and Quantitative Real‐Time PCR

Total RNAs from KO and control CD4 T‐cells (2 × 10^6^ cells for each condition) were isolated using the RNeasy Mini kit according to the manufacturer's instructions (Qiagen, 74104). The RNA was quantified by NanoDrop (Thermo Scientific) and then converted to cDNA using the iScript cDNA Synthesis kit (Bio‐Rad, 1708890). Quantitative real‐time PCR (qRT‐PCR) was performed using iTaq Universal SYBR Green Supermix (Bio‐Rad, 1725121) using a StepOnePlus real‐time PCR machine (Applied Biosystems). mRNA expressions of targeted genes were determined relative to a housekeeping gene *GAPDH*, and fold change was calculated using the 2^−ΔΔC^ *^t^* method. All primers used in this study are listed in Table S2.

### 5.5 Genotyping for Knockout Confirmations and Indels Within Targeted Exons

A total of 1 × 10^6^ cells were resuspended in 200 mL of PBS with 20 mL of proteinase K to lyse the cells, and genomic DNA from WT control and *Il6* KO CD4 T‐cells was extracted using the DNeasy Blood & Tissue kit (Qiagen, 69504), as instructed by the manufacturer [25]. The concentration of genomic DNA was determined by NanoDrop. The genomic reference sequence for *Il6* (NCBI GenBank accession no. NG_011640.1) was retrieved from the UCSC genome browser (https://genome.ucsc.edu/). The genomic region containing the *Il6* target sites was PCR amplified using genomic DNA from corresponding WT and *Il6* KO CD4 T‐cells with gene‐specific primer sets. The thermocycler setting consisted of one cycle of 95°C for 5 min, 35 cycles of 95°C for 15 s, 60°C for 15 s, and 72°C for 30 s, and one cycle of 72°C for 1 min. The PCR products were purified on 2% (w/v) GenPure LE agarose gel (ISC BioExpress, E‐3120‐500) and were eluted from the agarose gel using the QIAquick gel extraction kit (Qiagen, 28506) as described previously [25, 63]. The concentration of PCR DNA was quantified with a NanoDrop. A total of 500 ng of PCR DNA for each sample was used for Sanger's sequencing. To determine the nucleotide base pair insertions and/or deletions (indels) at the targeted sites in the *Il6* gene, nucleotide sequences were aligned with their respective KO, negative, and WT control sequences using the online EMBL‐EBI multiple sequence alignment tool (https://www.ebi.ac.uk/jdispatcher/msa).

### 5.6 T‐Cell Cultures and Ex Vivo Differentiation of Th17 Subsets

WT control and *Il6* KO CD4^+^ T‐cells (5 × 10^6^ of each) were resuspended at 1 × 10^6^ cells per milliliter in X‐VIVO 15 serum‐free media (Lonza, 04–418Q), followed by stimulation in various Th17 differentiation conditions (Media Alone/Th0, Th17a, Th17b, Th17c, and Th17d), as shown in Figure 1. The conditions were based on previous publications [15, 54, 64]. Media Alone/Th0: no cytokines/antibodies added; 2) Th17a: anti‐IL4 7 µg/mL BD554481, anti‐IFNγ 7 µg/mL BD554698, IL‐1β 10 ng/mL BD554602, TGF‐β1 10 ng/mL eBioscience 14–8348‐62; 3) Th17b: anti‐IL4 7 µg/mL, anti‐IFNγ 7 µg/mL, IL‐6 50 ng/mL BD550071, TGF‐β1 10 ng/mL eBioscience 14–8348‐62, 4) Th17c: anti‐IL4 7 µg/mL, anti‐IFNγ 7 µg/mL, IL‐6 50 ng/mL BD550071, IL‐1β 10 ng/mL BD554602 and TGF‐β1 10 ng/mL eBioscience 14–8348‐62; 5) Th17d: anti‐IL4 7 µg/mL, anti‐IFNγ 7 µg/mL, IL‐21 50 ng/mL Cell Sciences CRI172B and TGF‐β1 10 ng/mL eBioscience 14–8348‐62. IL6/STAT3 Pathway Blockade: CD4+ naive T‐cells were isolated, treated with human anti‐IL6R alpha antibody (R&D Systems, MAB227‐100) to block the IL‐6 receptor, and differentiated into Th17 subtypes in the presence of human recombinant cytokines as stated above. All the cultures were activated with 1 µg/mL each of fixed anti‐CD3 (eBioscience, 16‐0037‐85) and anti‐CD28 (eBioscience, 16‐0289‐85), as described previously [15, 48, 65] and incubated for 7 days at 37°C. Supernatants were aliquoted at day 7 for ELISAs, and cells were washed twice with phosphate‐buffered saline (PBS) for suppression assay cultures. In some experiments, an aliquot of cultured cells was used for intracellular cytokine staining to assess their state of differentiation. ELISAs were performed on supernatants as per the manufacturer's protocol (Invitrogen Human ELISA Kits for IL‐17A (BMS2017), IL‐6 (BMS213‐2), IL‐10 (BMS215INST), and GM‐CSF (BMS283). ELISA data were acquired on a BioTek Synergy H1 Hybrid Reader. Gen5 v2.09 was used for software analysis.

### 5.7 Flow Cytometric Suppression Assays

CD4+ T‐cells from the 7‐d differentiation were placed in flow cytometric suppression assays, as described previously [15, 27, 66]. Briefly, responder CD4^+^ T‐cells (1 × 10^6^) were stained with CFSE, followed by culture with 1 µg/mL each of fixed anti‐CD3 (eBioscience, 16‐0037‐85) and anti‐CD28 (eBioscience, 16‐0289‐85) in the presence or absence of ex vivo sorted autologous bulk CD8+ T‐cells. On day 7 of culture, cells were stained with anti‐CD4 PE‐Cy7 (BD Biosciences, 557852), anti‐CD3 Alexa Fluor 700 (BD Biosciences, 557943), anti‐CD8 BV786 (BD Biosciences, 563823), and anti‐CD25 Pacific Blue (BioLegend, 356129). Ab‐stained cells were flow cytometrically assessed for CD4 proliferating fraction (CFSE dilution). %proliferation and %suppression were calculated as described previously [15, 25, 27]. The flow cytometry gating strategy used to analyze CFSE‐stained CD4^+^ T cell proliferation is shown in Figure S9, outlining sequential gating of lymphocytes, single cells, CD4^+^ T lymphocytes, and CFSE low proliferating populations. All the flow cytometry‐related experiments were conducted following the guidelines for the use of flow cytometry and cell sorting in immunological studies [67].

### 5.8 Flow Cytometric Antibodies

The following antibodies were used for flow cytometry: for surface staining, BV711 mouse anti‐human CD3 (BD Pharmingen, 563725), APC mouse anti‐human CD4 (BD Pharmingen, 555349) and PE mouse anti‐human CD25 (BD Pharmingen, 555432), pacific blue anti‐human CD8 antibody (BioLegend, 344717) and for intracellular cytokine staining, PE‐Cy7 Mouse Anti‐Human IFN‐γ (BD Biosciences, 560741), IL‐17A monoclonal antibody (eBioscience, 17‐7179‐42), PE anti‐human IL‐6 antibody (BioLegend, 501107), Alexa Fluor 647 anti‐human IL‐1β Antibody (BioLegend, 508208), Brilliant Violet 42 anti‐human FOxP3 Antibody (BioLegend, 320123), PerCP/Cyanine5.5 anti‐STAT3 Phospho (Tyr705) antibody (BioLegend, 651022), Alexa Fluor 647 anti‐human IL‐12/IL‐23 p40 antibody (BioLegend, 501818), PE Mouse anti‐Human IL‐21 (BD Biosciences, 560463), PE Rat Anti‐human IL‐10 (BD Biosciences, 559330), PE anti‐human GM‐CSF antibody (BioLegend, 502306), human/mouse/rat Jak1 Alexa Fluor 488‐conjugated antibody (R&D systems, IC4260G). All cells were resuspended in 0.2% paraformaldehyde (Electron Microscopy Sciences, Hatfield, PA) for FACS analysis.

### 5.9 Statistical Analysis

GraphPad Prism (version 9.0.0) software was used for data analysis. Statistical significance was determined by an unpaired two‐tailed Student t‐test. All experiments were performed at least three times, and each experimental group included n2. Data are expressed as mean ± SEM. More than two groups were compared via one‐way ANOVA with a Bonferroni posttest for multiple comparisons. A *p*‐value <0.05 was considered significant.

### 5.10 Study Approval

All experiments were performed on PBMC obtained from deidentified LRS cones from healthy platelet donors at the University of Iowa DeGowin Blood Center, as approved by the University of Iowa Institutional Review Board.

## Author Contributions

C.V., M.C., and N.K. contributed to the study's conception and design. C.V., K.K., and M.C. performed the experiments and acquired and analyzed data. C.V. and N.K. organized the datasets. C.V. wrote the first draft of the manuscript. All authors contributed to the manuscript revision, read, and approved the submitted version.

## Conflicts of Interest

The authors declare no conflicts of interest.

## Supporting information




**Supporting File**: eji70150‐sup‐0001‐SuppMat.pdf.

## Data Availability

The data that support the findings of this study are available within this article, including supplementary materials.
