# Supplementary material for: CD4+ T‐Cell‐Intrinsic IL‐6 Is Critical for Th17 Differentiation and Dampened Responsiveness to CD8+ T Cell‐Mediated Suppression
Source: Eur J Immunol. 2026 Feb 23;56(2):e70150. doi: 10.1002/eji.70150 (PMC12929703; doi:10.1002/eji.70150)
Supplement: Supplementary file 1 — Supporting File: eji70150‐sup‐0001‐SuppMat.pdf. [file EJI-56-e70150-s001.pdf]

CD4⁺ T Cell–Intrinsic IL-6 Is Critical For Th17 Differentiation And Dampened Responsiveness To CD8⁺ T Cell-Mediated Suppression

Chakrapani Vemulawada, Kshitija Kale, Michael P. Crawford and Nitin J. Karandikar*

SUPPORTING INFORMATION

Pre-sort **Post-sort**

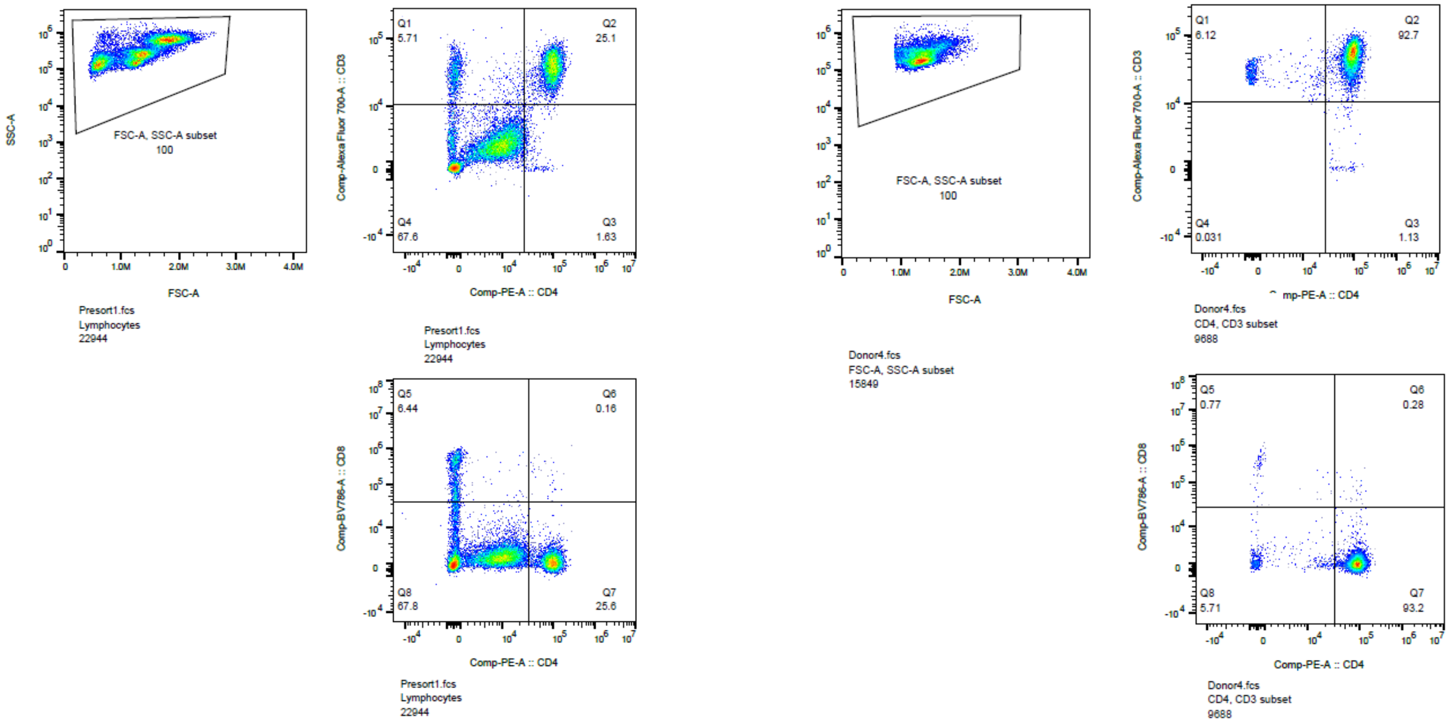

Figure S1. **Purity of naïve CD4⁺ T cells before and after isolation.**
Flow cytometry plots show ungated data (FSC/SSC), followed by CD3 vs CD4 and CD8 vs CD4 dotplots. Pre-sort (left): mixed PBMC populations with modest CD3⁺CD4⁺ frequency (~25%) and substantial CD8⁺ cells (~26%). Post-isolation (right; negative selection for naïve CD4⁺ T cells) shows highly enriched CD3⁺CD4⁺ cells (~93% of total events) with almost no non-lymphocytic populations and negligible CD8⁺ contamination (<1%). Representative of ≥10 donors.

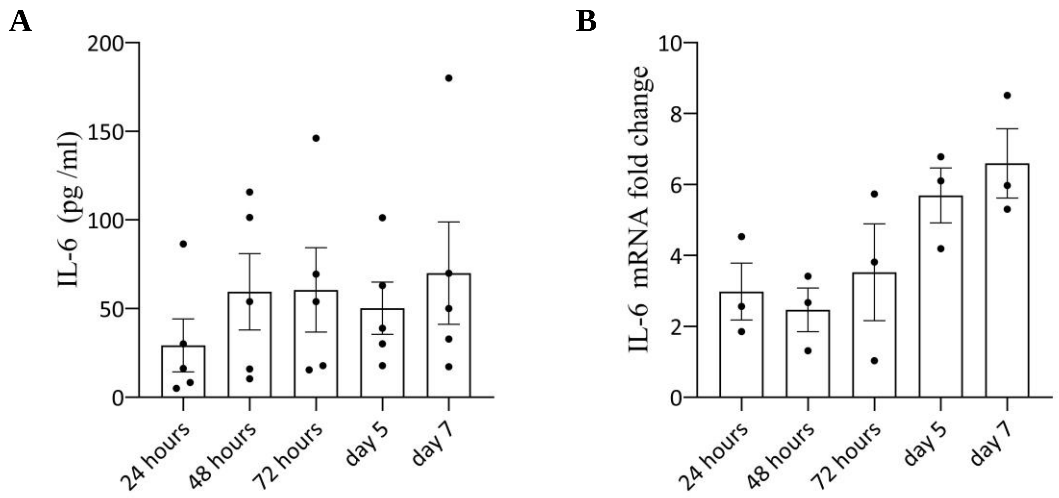

FIGURE S2. Ex vivo–derived CD4⁺ T cells produce IL-6 following activation. Naïve $CD4^{+}CD25^{-}CD45RO^{-}$ T cells from healthy donors were stimulated with plate-bound anti-CD3 and anti-CD28 and harvested at multiple time points (24 h, 48 h, 72 h, day 5, day 7). IL-6 protein in culture supernatants was measured by ELISA (A), and *Il6* mRNA expression was quantified by qRT-PCR relative to *Gapdh* with unstimulated cells as the reference (B).

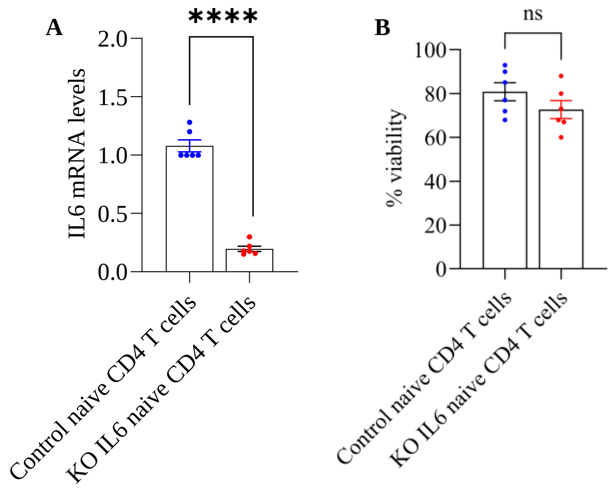

FIGURE S3. Nucleofection of *Il6* RNPs efficiently disrupts *Il6* in human naïve CD4^{+} T cells without affecting viability. At 24 h post-nucleofection, *Il6* mRNA expression was quantified by qPCR (A), and cell viability was assessed (B). Bar graphs show relative *Il6* mRNA levels and percentage of viable cells; each point represents an individual donor. Error bars indicate SEM, and statistical significance was determined using an unpaired Student's t-test.

Subset	Cytokines
Th0	Media alone
Th17.a	IL1β+TGF-β1
Th17.b	IL6+TGF-β1
Th17.c	IL1β+IL6+TGF-β1

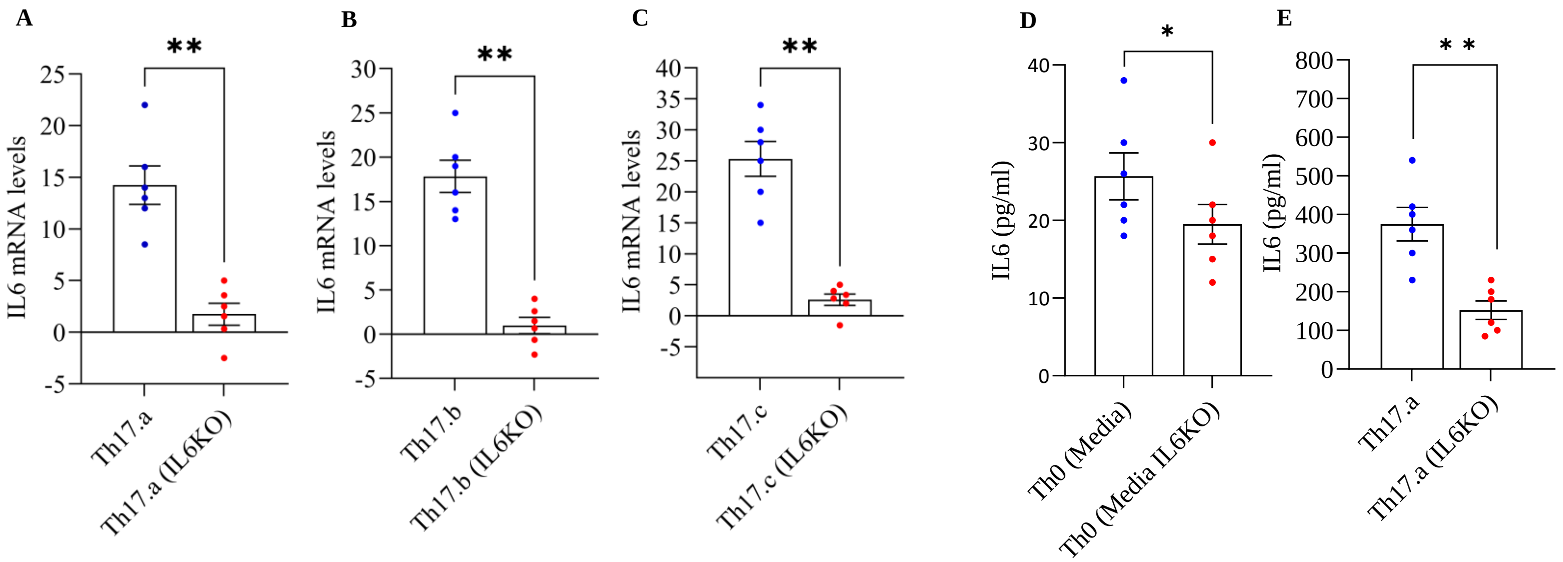

FIGURE S4. Confirmation of *Il6* deletion in T cells after 7 days of Th17 polarization. On day 7, *Il6* mRNA expression was measured in WT and *Il6* KO Th17 subsets (A–C). IL-6 protein levels in culture supernatants were assessed by ELISA in Th0 and Th17.a conditions (D, E); ELISA was not performed for Th17.b and Th17.c due to exogenous IL-6 in the media. Data are from three independent experiments (n = 6 per group); each point represents an individual donor. Error bars indicate SEM. Statistical analysis was performed using unpaired Student's t-test (*$p \leq 0.05$; **$p \leq 0.01$; ***$p \leq 0.001$; **$p \leq 0.0001$).

Subset	Cytokines
Th0	Media alone
Th17.a	IL1β+TGFβ1
Th17.b	IL6+TGF-β1
Th17.c	IL1β+IL6+TGF-β1
Th17.d	IL21+TGF-β1

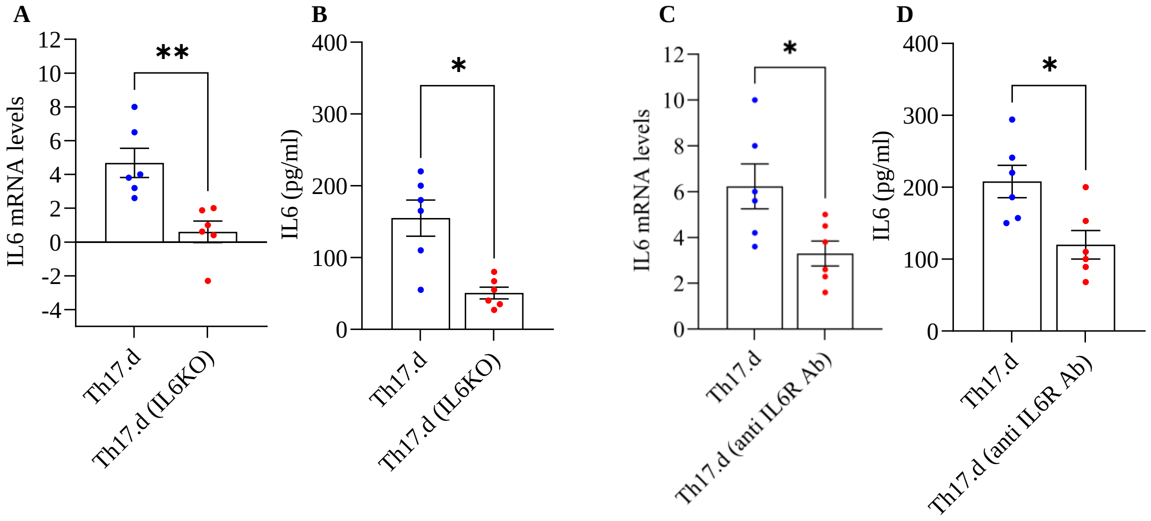

FIGURE S5. *Il6* mRNA and cytokine levels after *Il6* knockout or IL-6Rα blockade in Th17.d subsets. Naïve CD4^{+} T cells from WT, *Il6* KO, or IL6Rα–blocked conditions were polarized under Th17.d conditions for 7 days. *Il6* mRNA expression (A, C) and IL-6 protein levels by ELISA (B, D) were quantified. Data are from three independent experiments (n = 6 per group); each point represents an individual donor. Error bars indicate SEM. Statistical analysis was performed using unpaired Student's t-test (*p ≤ 0.05; **p ≤ 0.01; ***p ≤ 0.001; **p ≤ 0.0001).

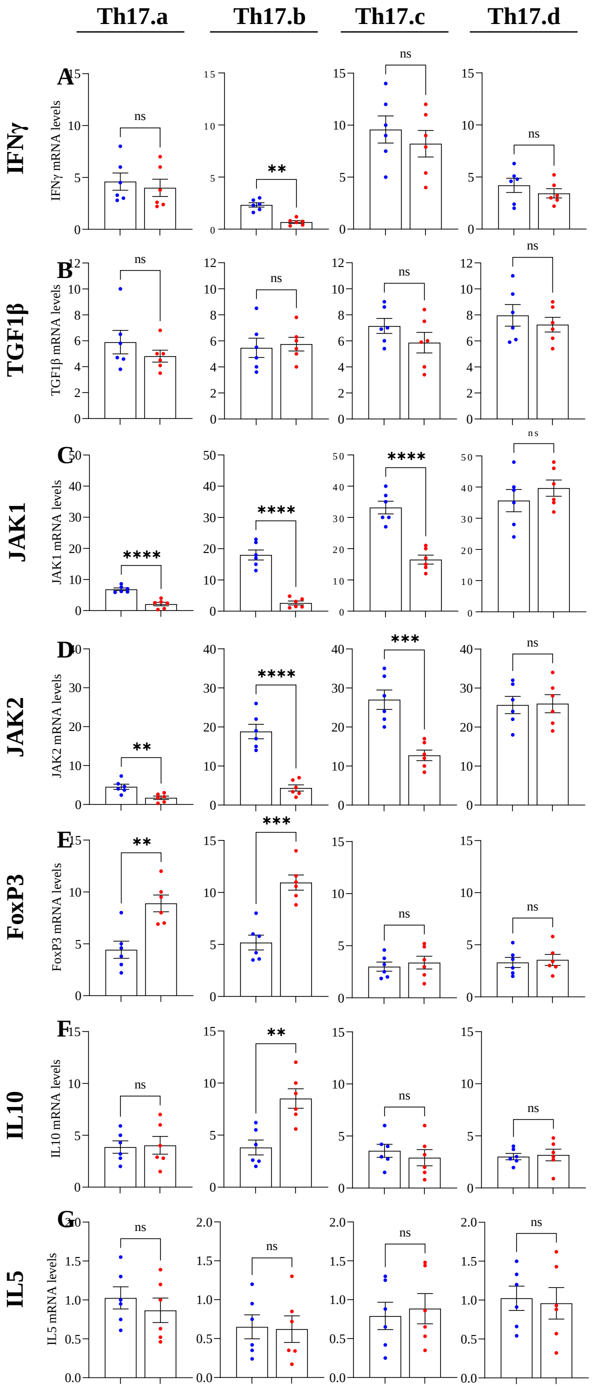

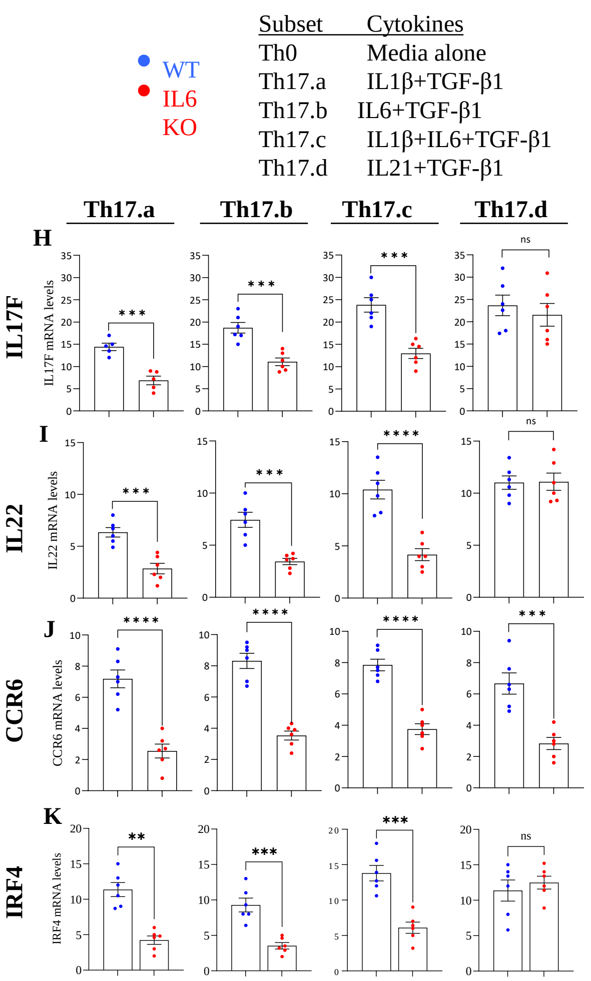

FIGURE S6. Differential regulation of cytokines and transcription factors in *Il6* KO Th17 subsets. Relative mRNA expression of *IFNγ*, *Tgfβ1*, *Jak1*, *Jak2*, *FoxP3*, *Il10*, and *Il5* (A–G), as well as *Il17F*, *Il22*, and *Irf4* (I–K), was measured in *Il6* KO Th17.a, Th17.b, Th17.c, and Th17.d subsets compared to WT controls. Data are from three independent experiments (n = 6 per group); each point represents an individual donor. Error bars indicate SEM. Statistical analysis was performed using unpaired Student's t-test (*$p \leq 0.05$; **$p \leq 0.01$; ***$p \leq 0.001$; **$p \leq 0.0001$).

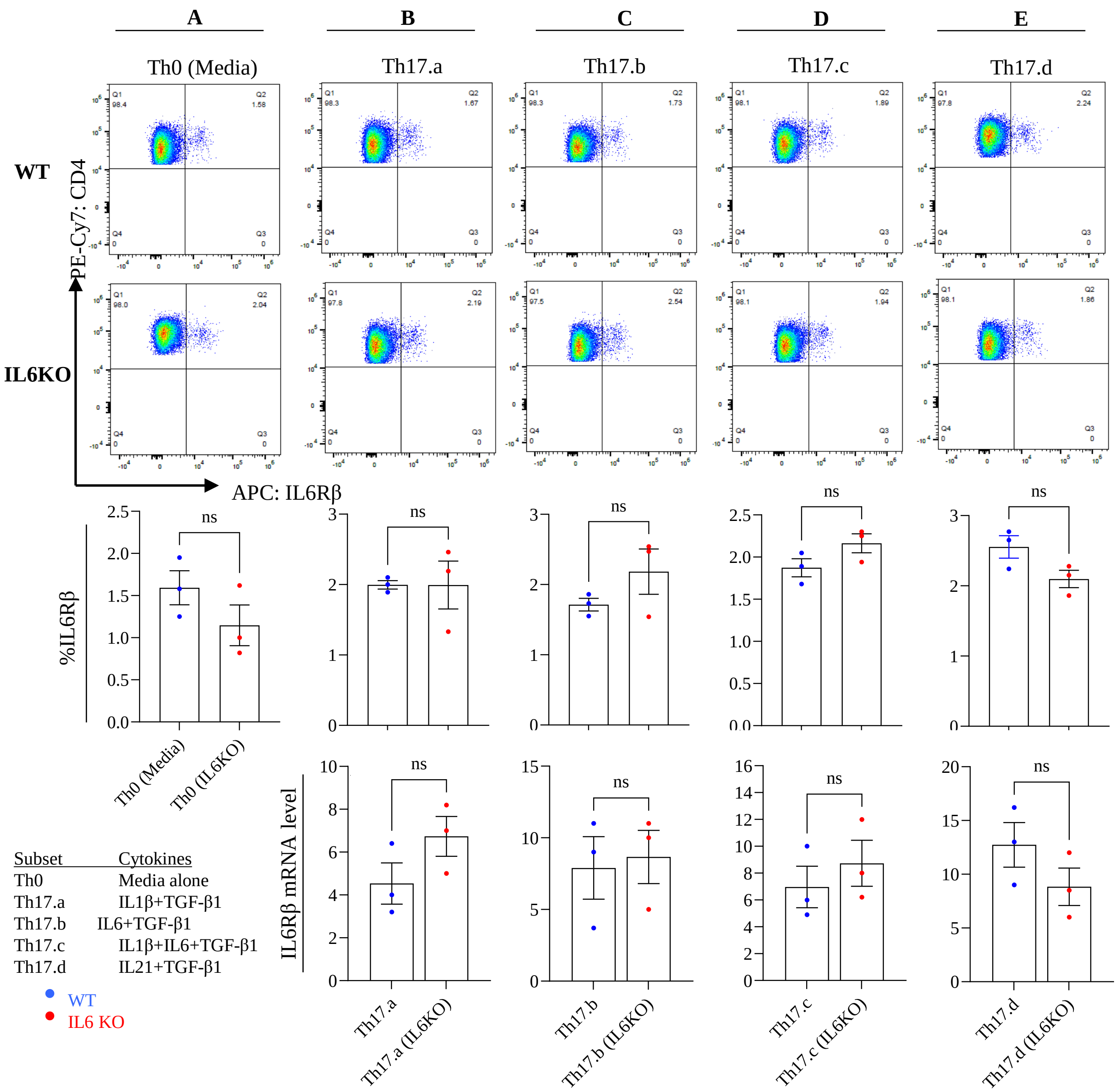

FIGURE S7. Endogenous *Il6* deletion does not alter *gp130* (IL-6Rβ) expression during Th17 differentiation. WT and *Il6* KO CD4^{+} T cells were cultured under the indicated Th17 conditions, and *gp130* expression was assessed by flow cytometry and qRT-PCR (A–E). Data are from three independent experiments (n = 3 per group); each point represents an individual donor. Error bars indicate SEM. Statistical analysis was performed using unpaired Student's t-test (*$p \leq 0.05$; **$p \leq 0.01$; ***$p \leq 0.001$; **$p \leq 0.0001$).

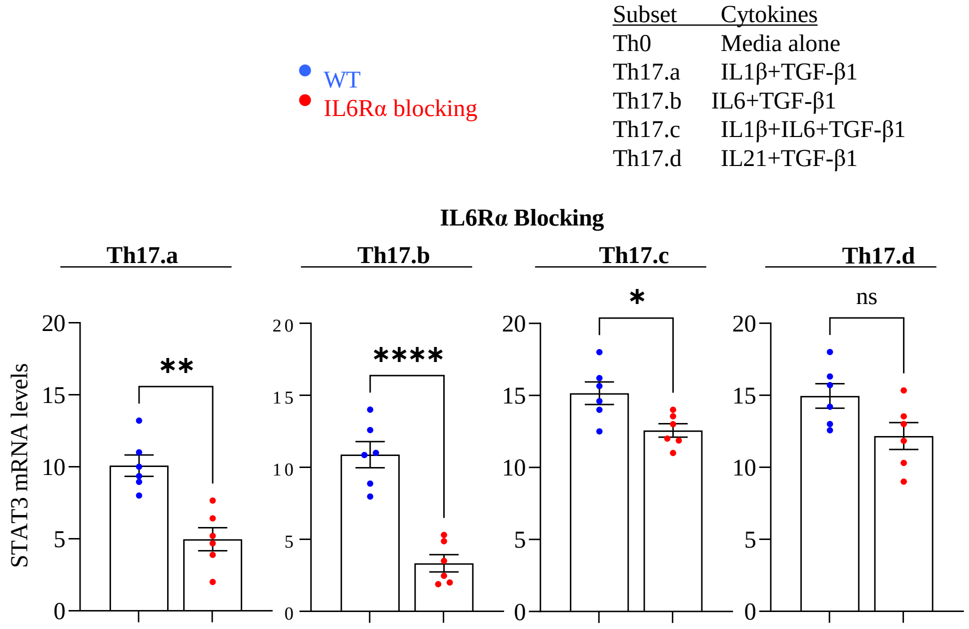

FIGURE S8. IL-6Rα blockade reduces *Stat3* expression in Th17 subsets. On day 7 of Th17 differentiation, *Stat3* mRNA levels were quantified in IL6Rα–blocked and WT Th17 subtypes. Data are from three independent experiments (n = 6 per group); each point represents an individual donor. Error bars indicate SEM. Statistical analysis was performed using unpaired Student's t-test (*$p \leq 0.05$; **$p \leq 0.01$; ***$p \leq 0.001$; **$p \leq 0.0001$).

Flow cytometry gating strategy

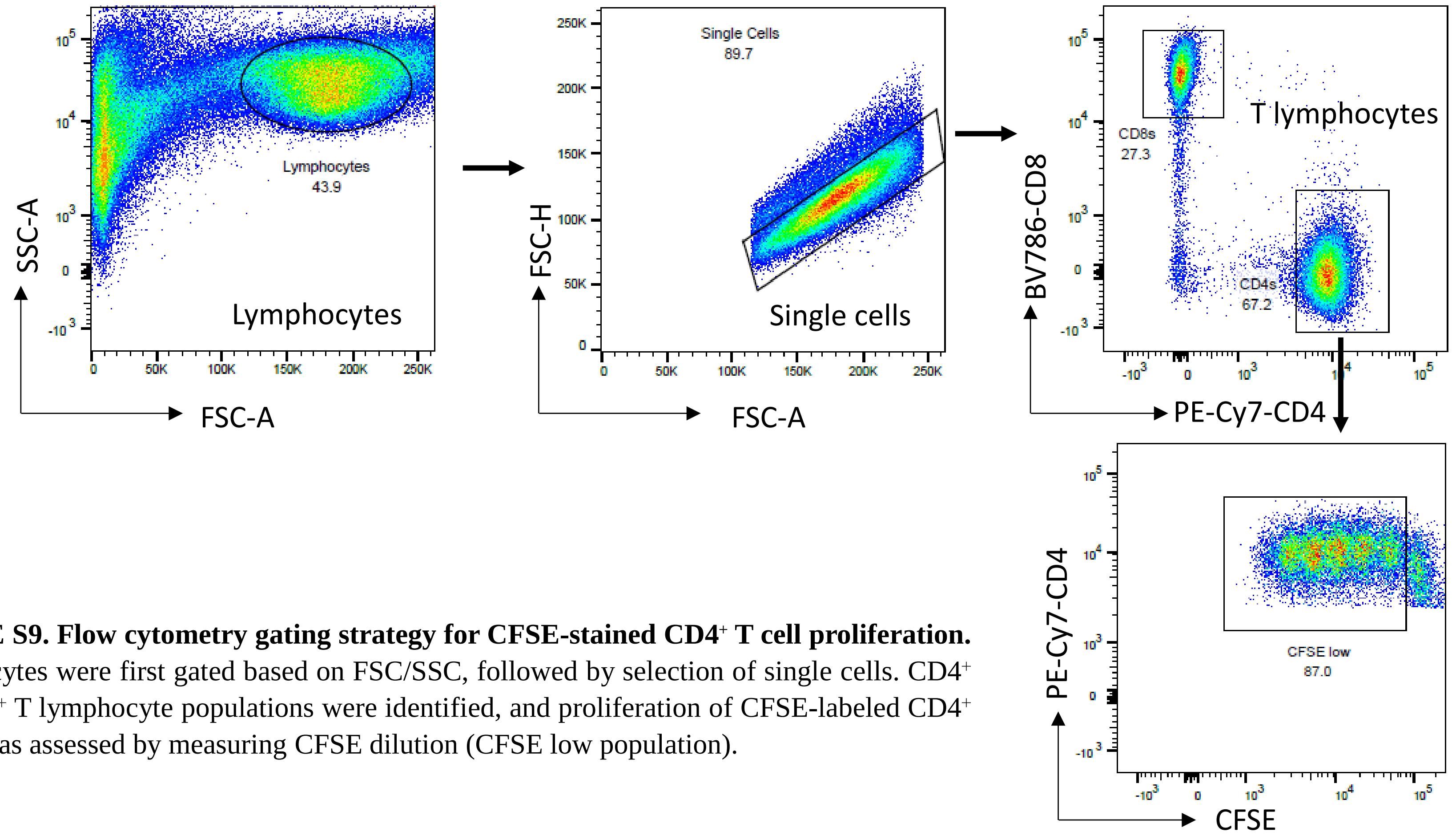

FIGURE S9. Flow cytometry gating strategy for CFSE-stained CD4⁺ T cell proliferation. Lymphocytes were first gated based on FSC/SSC, followed by selection of single cells. $CD4^+$ and $CD8^+$ T lymphocyte populations were identified, and proliferation of CFSE-labeled $CD4^+$ T cells was assessed by measuring CFSE dilution (CFSE low population).

CFSE stained-CD4 T cell proliferations

TABLE S1. gRNA sequences specific to human *Il6* gene used in this study, along with their targeting efficiencies, including on and off-target scores.

Gene	gRNA sequences (5'-3')	On/Off-target scores
Il6	AACGAAUUGACAAACAAAUU	72/68
	ACAUCUUUGGAAUCUUCUCC	78/73
	CCCUCCGGCACAGGCGCCUU	75/70

TABLE S2. Quantitative real-time PCR primers used in this study.

Gene	Forward Primer (5'—3')	Reverse Primer (5'-3')
Il6	TGGCTGAAAAAGATGGATGCT	GGCATTTGTGGTTGGGTCAG
Il6Rα	GACTGTGCACTTGCTGGTGGAT	ACTTCCTCACCAAGAGCACAGC
Il17A	CTGAGCCTGGAGGCCATAGT	TCTCTCAGGGTCCTCATTGC
Stat3	CAGCAGCTTGACACACGGTA	CTGCAGTCTGTAGAAGGCGT
RORγt	CCACAGAGACAGCACCGAG	TGCACCCCTCACAGGTGATA
T-bet	ACGCCGAGGGCTACCA	ACCAAGACCACGTCCACAAA
GM-CSF	AAACTTCCTGTGCAACCCAGA	CCTTGGTCCCTCCAAGATGAC
Il1β	TGAAGCTGATGGCCCTAAACAG	AGGTGCTCAGGTCATTCTCCT
gp130	GTACCGTGCATCGCACCTAT	GCTTCCTGGTCCATCAGCAT
IFNγ	GGCAAGGCTATGTGATTACAAGG	CATCAAGTGAAATAAACACACAACCC
Il21	CCAAGGTCAAGATCGCCACATG	TGGAGCTGGCAGAAATTCAGGG
Tgfβ1	TGTGGCTACTGGTGCTGAC	CGCAGCTTGGACAGGATCT
Jak1	GAGACAGGTCTCCCACAAACAC	GTGGTAAGGACATCGCTTTTCCG
Jak2	CCAGATGGAAACTGTTCGCTCAG	GAGGTTGGTACATCAGAAACACC
FoxP3	GGCACAATGTCTCCTCCAGAGA	CAGATGAAGCCTTGGTCAGTGC
Il10	TCTCCGAGATGCCTTCAGCAGA	TCAGACAAGGCTTGGCAACCCA
Il5	TGAGGATGCTTCTGCATTTGAG	TGGCTATCAGCAGAGTTCGATG
Il17F	AACCAGCGCGTTTCCATGTCAC	GAGCATTGATGCAGCCCAAGTTC
Il22	GTTCCAGCCTTATATGCAGGAGG	GCACATTCCTCTGGATATGCAGG
Ccr6	CTGAACCCTGTGCTCTACGCTT	CACAGGAGAAGCCTGAGGACTT
Irf4	GAACGAGGAGAAGAGCATCTTCC	CGATGCCTTCTCGGAACTTTCC
Gapdh	GTCTCCTCTGACTTCAACAGCG	ACCACCCTGTTGCTGTAGCCAA
